# *Helicobacter pylori* Infection: Current Status and Future Prospects on Diagnostic, Therapeutic and Control Challenges

**DOI:** 10.3390/antibiotics12020191

**Published:** 2023-01-17

**Authors:** Ayman Elbehiry, Eman Marzouk, Musaad Aldubaib, Adil Abalkhail, Sulaiman Anagreyyah, Nuha Anajirih, Abdulaziz M. Almuzaini, Mohammed Rawway, Abdulmajeed Alfadhel, Abdelmaged Draz, Akram Abu-Okail

**Affiliations:** 1 Department of Public Health, College of Public Health and Health Informatics, Qassim University, Al Bukayriyah 52741, Saudi Arabia; 2 Department of Bacteriology, Mycology and Immunology, Faculty of Veterinary Medicine, University of Sadat City, Sadat City 32511, Egypt; 3 Department of Veterinary Medicine, College of Agriculture and Veterinary Medicine, Qassim University, Buraydah 52571, Saudi Arabia; 4 Department of Preventive Medicine, King Fahad Armed Hospital, Jeddah 23311, Saudi Arabia; 5 Medical Emergency Services Department, Faculty of Health Sciences, Umm Al-Qura University, Al-Qunfudah P.O. Box 1109, Saudi Arabia; 6 Biology Department, College of Science, Jouf University, Sakaka 42421, Saudi Arabia; 7 Botany and Microbiology Department, Faculty of Science, AL-Azhar University, Assiut 71524, Egypt; 8 Performance Excellence and Quality, Qassim Health Cluster, Buraydah 52367, Saudi Arabia

**Keywords:** *Helicobacter pylori*, history, pathogenesis, diagnosis, therapy, prevention

## Abstract

*Helicobacter pylori* (*H. pylori*) infection, which affects approximately half of the world’s population, remains a serious public health problem. As *H. pylori* infection leads to a number of gastric pathologies, including inflammation, gastroduodenal ulcers, and malignancies, early detection and treatment are crucial to preventing the spread of the infection. Multiple extragastric complications, such as iron deficiency anaemia, immune thrombocytopenic purpura, vitamin B12 deficiency, diabetes mellitus, cardiovascular diseases, and certain neurological disorders, have also been linked to *H. pylori* infection. An awareness of *H. pylori* and associated health hazards is necessary to minimize or even eradicate the infection. Therefore, there is an urgent need to raise the standards for the currently employed diagnostic, eradication, alternative treatment strategies. In addition, a brief overview of traditional and cutting-edge approaches that have proven effective in identifying and managing *H. pylori* is needed. Based on the test and laboratory equipment available and patient clinical characteristics, the optimal diagnostic approach requires weighing several factors. The pathophysiology and pathogenic mechanisms of *H. pylori* should also be studied, focusing more on the infection-causing virulence factors of this bacterium. Accordingly, this review aims to demonstrate the various diagnostic, pathophysiological, therapeutic, and eradication tactics available for *H. pylori*, emphasizing both their advantages and disadvantages. Invasive methods (such as quick urease testing, biopsy, or culture) or noninvasive methods (such as breath tests, stool investigations, or serological tests) can be used. We also present the most recent worldwide recommendations along with scientific evidence for treating *H. pylori*. In addition to the current antibiotic regimens, alternative therapies may also be considered. It is imperative to eradicate the infections caused by *H. pylori* as soon as possible to prevent problems and the development of stomach cancer. In conclusion, significant advances have been made in identifying and treating *H. pylori*. To improve eradication rates, peptide mass fingerprinting can be used as a diagnostic tool, and vaccines can also eliminate the infection.

## 1. Introduction

It was first observed in the late nineteenth century that *Helicobacter pylori* (*H. pylori*), a highly mobile gram-negative, distinctively twisted bacterium, was present in the gastrointestinal system [1,2]. The researchers who demonstrated that *H. pylori* can cause gastritis received the Nobel Prize in 2005 due to the wide-ranging implications of their discovery [2]. Because the stomach was assumed to be a sterile organ where bacteria could not grow due to the low pH, the bacteria were presumed to have been orally ingested rather than being gastric inhabitants. *H. pylori*, however, has been linked with several digestive illnesses manifesting as indigestion since it was first discovered in the early 1980s by Warren and Marshall [3,4,5].

*H. pylori* is typically associated with chronic active gastroenteritis, and the bacteria lives in the glands beneath the mucosal surface [6]. There is a significant relationship between *H. pylori* infection and stomach cancer, peptic ulcer illness, and gastric mucosal lymphoid tissue lymphoma [3,5]. A study conducted by Shatila and Thomas [7] indicated that *H. pylori* infection can result in stomach carcinoma and mucosa-associated lymphoid tissue lymphoma in 90% of cases. Furthermore, *H. pylori* infection is closely related to stomach ulcers (up to 80% of cases) and duodenal ulcers (in approximately 90% of cases). There is also a close relationship between *H. pylori* infection and duodenal ulcers (present in 80% of cases), stomach ulcers (up to 80% of cases), and carcinomas [8]. As part of its 2014 recommendations, the World Health Organization (WHO) urged the eradication of *H. pylori* to reduce stomach cancer fatalities worldwide. Among the potential hazards to public health and the environment are bacterial strains of *H. pylori* that are clarithromycin-resistant [9].

Approximately half of the worldwide population is colonized by *H. pylori*, and the colonized population is incredibly widespread [10,11]. There is no clear way to explain how this bacterium is spread, but oral or faecal exposure leading to person-to-person transfer is thought to be the dominant method [11]. *H. pylori* is more commonly found in Asia, Latin America, and Africa than in North America and Oceania, where it may be found in only 24% of the population [12,13]. Among *H. pylori*-infected individuals, 34.7% live in industrialized nations, while 50.8% live in resource-poor nations [14], and most contemporary studies indicate that *H. pylori* infection incidence has been steadily decreasing. Despite this pattern, there has been an alarming increase in antibiotic-resistant strains of *H. pylori* [15]. Children are usually asymptomatic transmitters of infections who later develop signs as adults. However, it is true that the vast majority of infected people do not actually exhibit symptoms of *H. pylori* infection [16]. During outbreaks, the number of people affected by *H. pylori* infection varies between 85 and 95% in poor nations and between 30 and 50% in industrialized nations [17,18,19]. Unfortunately, the exact method by which *H. pylori* is transmitted is unknown. There are, however, reports that it is distributed through the faecal–oral and/or oral-to-oral routes. Drinking water and food tainted with this pathogen are associated with this form of spreading [11,16]. Infections are more likely to occur as a result of poor hygiene, insufficient nutrition, and geographical variances [20], whereas the development of some virulent factors allows *H. pylori* to persist at a lower pH level. Since the bacterium cannot produce acid itself, the urease enzyme neutralizes gastric acid [20].

Since *H. pylori* is linked to a number of gastric diseases, such as gastroenteritis, gastroduodenal ulcers, and even stomach carcinoma, it is critical to diagnose and treat infection with this pathogen early and effectively to prevent it from spreading [21]. *H. pylori* infection diagnostic tests are classified into two broad categories: invasive procedures (gastric biopsy, endoscopy-mediated) and noninvasive procedures (liquid biopsy). Nonendoscopic tests include the antigen detection test and the urea breath test for identifying vigorous *H. pylori* infections [22]. It is also possible to test for urease in stomach samples obtained during endoscopy, with a sensitivity and specificity of approximately 90% and 95%, respectively [23]. *H. pylori* infections are diagnosed histopathologically with 95% and 98% sensitivity and specificity, respectively. Antigen stool tests represent the most economical diagnostic approach currently available in areas with low-to-moderate *H. pylori* prevalence. Despite their high specificity and low sensitivity, prompt monoclonal immunochromatographic antigen stool tests are limited in their utility [24]. It is often possible to obtain fast results from PCR stool tests from commercial sources [25]. Serology-based diagnostics are ineffective in identifying current *H. pylori* infections because *H. pylori* antibodies linger even after the infection has been eradicated [4]. The recognition of active infections with *H. pylori* by endoscopy, along with noninvasive diagnostic tests (e.g., urea breath and antigen stool tests), may be less sensitive if bismuth or antimicrobials are taken within one to two weeks of the test.

It is still unclear and controversial what role this pathogen plays in stomach disorders. *H. pylori* causes passive inflammation inside the gastric epithelium and alters signal transduction pathways that serve as a platform for pathogenesis, but it also develops antimicrobial resistance via genetic changes and biofilm development [26,27]. It is also important to note that strain variation plays a role in the virulence of *H. pylori*, in addition to a number of other factors. The development of specific virulence genes facilitates the interaction between bacteria and hosts [28]. In a previous study conducted by Palamides et al. [29], different isolates of *H. pylori* had different pathogenicity and were associated with different prognoses. Despite the difficulty of removing *H. pylori*, it has been somewhat successful to date. It is crucial to identify the virulence and pathogenic pathways of *H. pylori* to develop effective methods to combat *H. pylori* infection [19,30]. From these virulence mechanisms, therapeutic approaches may be derived. For new medications and vaccines to be developed, it is therefore important to recognize exactly how virulence factors affect *H. pylori* pathogenicity. Throughout this article, we discuss the characteristics and clinical features of *H. pylori* infection, and we provide a brief summary of conventional and cutting-edge identification techniques that are effective for identifying and treating infections with this pathogen. A review of the vaccination strategies for and pathogenicity of *H. pylori* is also included.

## 2. The Historical Background of *H. pylori*

It is estimated that *H. pylori* left Africa approximately 60,000 years ago within an infected individual [31]. Previously, *H. pylori* had been found in contemporary animals before people migrated out of Africa and was eventually found in humans [32]. As early as 1982, doctors Barry Marshall and Robin Warren of Perth, Western Australia, discovered *H. pylori* in patients suffering from inflammation and ulcers in their gastric mucosa. A widespread belief at the time was that germs cannot survive in the acidic environment of the stomach. As a result of Marshall and Warren’s discovery, Physiology’s Nobel Prize was awarded to them in 2005. Marshall and Warren’s study was the first to find spiral-shaped bacteria in the stomach wall; however, German researchers were not able to cultivate them, so their findings were ignored [33]. According to some modest studies conducted in the early twentieth century, most people suffering from gastric ulcers and gastric cancer had bent rods in their stomachs. It is noteworthy that an American investigation of 1180 gastric samples reported in 1954 did not find the bacteria. This led to a decline in enthusiasm for research on these bacteria [34].

Since the 1970s, when bacteria in the guts of stomach ulcer patients were visualized, curiosity about bacterial roles in gut illness has been renewed [35]. Likewise, Robin Warren and Barry Marshall had seen the bacteria in 1979, and they studied it together beginning in 1981. They saw colonies only after accidentally leaving petri dishes incubating for five days during the Easter weekend in 1982, after numerous failed attempts to cultivate stomach bacteria. Unlike earlier researchers, Warren and Marshall maintained that the majority of gastritis cases and peptic ulcers are caused by bacterial infections rather than stress or salty foods [36]. It was initially believed that gastritis and ulcers were not related, but after several years, numerous teams of scholars confirmed this link [37]. To demonstrate that *H. pylori* was the cause of his gastritis and not simply a by-product, Marshall swallowed some cultured *H. pylori*. He began feeling ill with nausea and vomiting a few days later. He underwent endoscopy 10 days after inoculation, which showed signs of gastritis and *H. pylori* in his stomach. As a result of these findings, *H. pylori* was determined to be the causal agent.

According to Marshall and Warren, many cases of gastritis can be successfully treated with antimicrobials. In 1994, the National Institutes of Health (NIH) suggested including antimicrobials in the treatment protocol for gastric and duodenal ulcers caused by *H. pylori* [38]. Many papers have been published since 1997 detailing the pathophysiology, immunology, and pathogenicity of *H. pylori*. Depending on the region, *H. pylori* infection varies, with developing countries bearing the heaviest burden [39]. Several external factors, including food, carcinogen exposure, excessive alcohol consumption, and tobacco, can contribute to *H. pylori* development. *H. pylori* infections after invasion can also be influenced by the persistence of the bacteria as well as their pathogenicity [40,41].

## 3. The Virulence and Pathogenic Pathways of *H. pylori*

**Figure 1:**
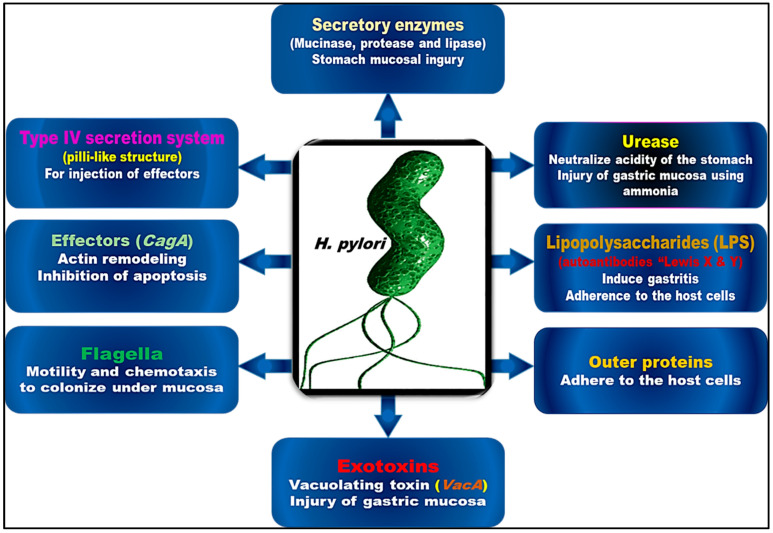
Virulence factors of *H. pylori*: their role in pathogenesis and effectiveness on host cells.

*H. pylori* infection is classified in three stages: the colonization of the stomach mucosa, the consequent immune response, and disease development. Figure 1 illustrates several virulence factors of *H. pylori* that contribute to its pathogenicity and effects on host cells. The bacterium floats in the direction of the epithelial membrane when it enters the stomach, taking advantage of areas of the stomach wall that are injured [42,43]. It uses Tlp receptors, mainly TlpB, to regulate flagellar motion based on chemical messengers in the cell environment [44]. Reactive oxygen species, as well as urea, gastric acid, lactate, and gastric acid, serve as signals for these receptors; urea is a key factor in microbial invasion [44]. There are also unknown molecules that may play a role in this mechanism [45]. *H. pylori* uses urease to defend itself against the acidic medium around it. Urea is converted into ammonia and other beneficial compounds by urease, which raises the pH of the microenvironment while protecting the bacterium from the acid in the stomach. In the presence of this barrier, the mucosal gel lining the stomach wall becomes less viscous, allowing the bacteria to travel through the mucus towards the gastric pits in which they will eventually colonize [45,46].

For bacteria to adhere to stomach epithelial cells, Lewis antigens and multiple components must interact in a complex manner. A Lewis (Le) antigen is a glycoprotein found on the surface of cells that is attached to a selectin on a target cell, facilitating attachment between them [47,48]. Lewis-like antigens are expressed in the lipopolysaccharide (LPS) component of the *H. pylori* cell wall, with Le^X^, in particular, showing modest adhesion functions [49,50]. Conversely, the outer membrane proteins (OMPs) of the bacterial cell wall serve as signalling pathways that allow the host Lewis antigens to attach to the OMPs. *H. pylori* OMPs can be divided into five genetic groups [51,52], of which the outside membrane and the OMP play the most important role [53,54]. Blood antigen-binding adhesion (*BabA*) and sialic acid-binding adhesion (*SabA*) are two OMPs in the *H. pylori* outer membrane family that are well investigated [52]. Cell attachment is stimulated by *SabA* binding to sialylated Le^X^ (sLe^X^), while cell-to-cell binding is facilitated by *BabA* attaching to host Le^B^ [55,56]. Additionally, *SabA* promotes neutrophil activation by attaching to sLe^X^ and triggering G-protein-coupled signalling [57]. Infection with *H. pylori* and stomach inflammation are associated with higher levels of sLe^X^, suggesting that SabA might promote rather than strengthen and sustain adherence. Mucin receptors (MUC5a and MUC1) are the primary sites of action for these OMPs, which are both capable of hastening and preventing infection [58]. Although *BabA* and *SabA* are the primary adhesins, there are also other outer membrane proteins, such as Helicobacter outer inflammatory protein A (*OipA*) and Helicobacter outer membrane proteins Q and Z, that enhance *H. pylori* adhesion and inflammation by increasing the expression of virulence genes and cytokine production [52].

*H. pylori* also has virulence traits that enable it to change the surroundings for its own benefit in addition to weapons that directly affect host cells and adhesive components that let it adhere to its host [59]. Several virulence factors related to pathogenicity aid adhesion as well. There is evidence that the *BabA*-Le^B^ interaction activates the type IV secretion system (T4SS), which is a pilus-like structure that permits the transfer of regulatory proteins such as cytotoxin-associated gene A (*CagA*) and vacuolating cytotoxin A (*VacA*) [56]. Direct attachment of epithelial cells to integrin-1 is required for *CagA* to attach to and disrupt the signal transduction pathways of *H. pylori* [60]. The *VacA* protein directly interacts with a number of targets and has a number of downstream effects. In persistent infections, the *VacA* protein plays a crucial role in avoiding the immune response. It is mainly a pore-forming toxin that abruptly kills host cells. The latter function is accomplished by hindering phagocytosis and creating cytoplasmic vacuoles in the host cell where *H. pylori* can live. Toxins are the most important virulence factors of *H. pylori* and are essential to its virulence. In most strains of *H. pylori*, *VacA* is produced, whereas *CagA* is present in only a few strains. In fact, *CagA* positivity has been linked to more serious infections, poorer treatment outcomes and a greater likelihood of developing cancer in the future [26]. Although these two proteins are the main contributors to the pathogenesis of *H. pylori* infection, other proteins also play an integral role in attachment, immune evasion, and inflammation [19,26].

## 4. *H. pylori* Infection and Extraintestinal Disorders

There are several illnesses caused primarily by *H. pylori* infection, such as chronic gastritis, gastric ulcers, duodenal ulcers, and gastric adenocarcinomas [3,61,62]. There can be a considerable burden placed on the diagnosis and treatment of *H. pylori* infection due to the presence of extraintestinal symptoms [63]. Several papers related to the topic of extragastroduodenal disorders of *H. pylori* infection have been published in recent decades, including haematological, metabolic, cardiovascular, neurodegenerative, and allergy illnesses [62,63,64,65,66,67,68]. In most cases, extraintestinal manifestations are due to systemic subclinical inflammation caused by *H. pylori*, which is why early eradication may limit any unfavourable effects associated with the onset of these symptoms [63]. Thus, it was suggested that *H. pylori* infection may contribute to iron deficiency anaemia, thrombocytopenia, failure to thrive, diabetes mellitus, body mass index, cardiovascular disorders, and several neurological disorders [63].

Iron deficiency is considered to be one of the most prevalent dietary deficiencies, affecting approximately 500 million people worldwide [69]. Because *H. pylori* is one of the most prevalent bacteria in the world, it is not surprising that researchers have focused on finding a causal relationship between *H. pylori* infection and iron deficiency. Blecker et al. first identified the association between *H. pylori* and iron deficiency anaemia in 1991 [70]. The patient, a 15-year-old Belgian, had chronic active haemorrhagic gastritis caused by *H. pylori* and iron deficiency anaemia, which completely disappeared, without iron supplementation, when *H. pylori* was eradicated [70,71,72].

A study conducted by Gasbarrini et al. [73] in 1998 demonstrated a significant platelet count increase following *H. pylori* eradication. Furthermore, Garcia Perez et al. [74] observed in a patient who had developed chronic immunological thrombocytopenic purpura after removing this bacterium that the platelet count returned to normal once the bacterium was eradicated. Stasi et al. [75] also reported parallel findings that 50% of adults with mild immune thrombocytopenic purpura were found to have prolonged platelet responses after the eradication of *H. pylori*. However, several investigations have not found a correlation between the severity of immune thrombocytopenic purpura and this infection [76].

In 1984, when O’Connor et al. discovered Campylobacter-like organisms in patients with type A gastritis associated with pernicious anaemia, they proposed that vitamin B12 deficiency contributes to *H. pylori* infection [77]. Based on several studies that have been carried out and that established a link between *H. pylori* infection and vitamin B12 malabsorption, it has been found that more than half (67.4%) of patients with *H. pylori* infection also have this deficiency [78,79]. According to a study conducted on patients with this condition, *H. pylori* infection and vitamin B12 deficiency are significantly correlated [80]. There has been some evidence indicating that *H. pylori* infection at the carotid plaque level may facilitate the progression of atherosclerosis, leading to ischaemic stroke, especially in patients with strains containing the *CagA* gene. Moreover, the authors noted that ischaemic cerebrovascular stroke can also be related to *H. pylori* infections in patients with ischaemic cerebrovascular strokes.

A number of studies have also suggested that *H. pylori* may be associated with insulin resistance, diabetes mellitus, and metabolic syndrome [81]. The results of some studies, however, found that *H. pylori* was more common in patients with diabetes mellitus than in those without diabetes mellitus. Other studies [82,83,84] were not able to find any correlation between the two conditions. Accordingly, Nasif et al. demonstrated that patients with type 2 diabetes mellitus were more likely to harbour *H. pylori* than those without diabetes mellitus [85]. There is strong evidence to support the fact that eradicating *H. pylori* infection reduces the risk of diabetes development [86]. Moreover, Song and colleagues suggested that diabetic patients with *H. pylori* infection may require more rigorous eradication therapy, especially those with poorer levels of glycaemic control and a higher body mass index than those without *H. pylori* infection [87].

The findings of a recent study, however, have suggested that there might be a connection between *H. pylori* infection and certain neurological disorders, such as Parkinson’s disease and Alzheimer’s disease. However, the validity of these findings is still debatable. According to one theory, the development of Parkinson’s disease may be related to the damage that *H. pylori* can cause to dopaminergic cells in the nervous system [88]. A study conducted by Tan et al. reported that *H. pylori* may make the motor symptoms of Parkinson’s disease patients more serious as a result of gastrointestinal infection [89]. It is even more difficult to determine the effects of elimination. Therefore, some researchers have indicated that the elimination of *H. pylori* in Parkinson’s disease patients led to improvements in the efficacy of levodopa, clinical symptoms, and quality of life [69]. A number of studies have failed, however, to find a causal relationship between the eradication of this bacterium and the clinical outcomes of Parkinson’s disease [90].

## 5. Diagnostic Approaches for *H. pylori*

**Figure 2:**
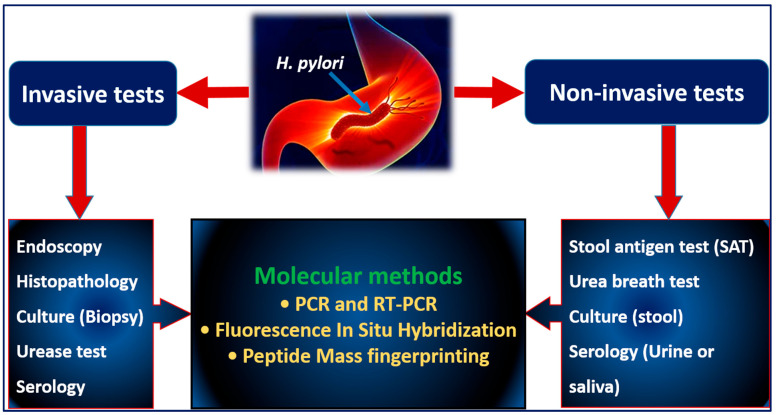
Invasive and noninvasive *H. pylori* diagnostic tools.

A variety of diagnostic approaches are available, each of which has its own advantages and disadvantages [21]. There are several types of tests, the selection of which is based on the availability of the tests, the tools available, and the medical needs of the hospital. Diagnostic testing can be performed with both invasive and noninvasive techniques. Examples of noninvasive techniques are serological, stool antigen, and breath tests, whereas invasive techniques include endoscopy, histopathological analyses, quick urea tests, cultures, and PCR tests (Figure 2). In addition to invasive and noninvasive procedures, molecular tools such as PCR, real-time PCR, fluorescence in situ hybridization, and peptide mass fingerprinting are commonly used.

### 5.1. Invasive Tests

Invasive techniques are used for obtaining biopsy samples from the antrum and bottom of the stomach and duodenum during endoscopy. Histopathological examinations and microaerophilic cultivation can both be performed on the specimens.

#### 5.1.1. Endoscopy

Endoscopy is considered to be among the more invasive techniques for the diagnosis of peptic ulcer disease. This technique has been established to be effective in patients who do not exhibit any new worrisome symptoms or signs of any digestive disorder [21]. Several distinct characteristics are associated with gastritis, including inflammation, shrinkage, and intestinal metaplasia, which makes diagnosis difficult. Recent techniques for endoscopic imaging combine blue laser imaging (BLI) with linked colour imaging (LCI). All of the investigation results indicate that blue laser magnifying endoscopy and LCI greatly improves the endoscopic method; nonetheless, BLI is still the most accurate technique for determining tumour formation [91,92]. A routine endoscopic evaluation involves obtaining specimens that can generate valuable information for more informative procedures, e.g., *H. pylori* cultures, fast urea tests, or histopathological analysis, the gold standard of diagnosis [93]. To assess *H. pylori* gastritis, it is necessary to take at least six biopsy samples from the centre of the stomach region, the large and small curvatures and the antrum of the stomach. It is necessary to perform obtain more biopsy samples of lesions that are worrisome, bleeding ulcers, and localized lesions. Endoscopes with magnifying capabilities allow for significant improvements in collecting biopsy samples, for example, with narrowband endoscopy or blue light endoscopy [94].

#### 5.1.2. Histopathology

Infection with *H. pylori* was first identified by histopathological examinations, and they remain the most common method of detection. Several factors affect the diagnostic precision of histopathology, including specimen position and thickness, staining techniques, proton pump inhibitors, antimicrobials, and the pathologist reviewing the specimens [95,96]. Biopsy samples taken from different sites; their size, quantity, and colouring; and the use of medications such as proton pump inhibitors all play a role in producing false-negative results. According to the Maastricht guidelines, patients should cease proton pump inhibitor therapy at least two weeks undergoing biopsy. When a biopsy is performed, numerous samples are taken from the distal and middle regions of the stomach. Haematoxylin eosin (H&E) staining, Giemsa staining, *H. pylori* silver staining, and immunohistochemistry are widely used staining methods in practice [21]. Most clinical settings utilize H&E and Giemsa staining. It is usually not possible to perform immunohistochemistry, although it provides the most obvious and accurate staining [43]. Despite its decent sensitivity, H&E staining has a low specificity of only 75% compared with Giemsa staining (90%) and immunohistochemistry (100%) for detecting *H. pylori* [45,46]. Overall, Giemsa staining has a higher specificity and lower false-positive rate but a lower sensitivity than H&E staining. This rate can be further reduced by using IHC in laboratory work [48]. Histochemical staining of a gastric sample is sufficient to diagnose *H. pylori* infection in most patients. Whenever the histochemical method is unsuccessful in demonstrating the presence of *H. pylori* in samples from patients with persistent (active) gastritis, immunohistochemistry should be used to identify the organism. The fluorescent nucleic acid peptide in situ hybridization method, which identifies unreported *H. pylori* types, is fast and reasonably priced and guarantees 100% accuracy. As a drawback, it requires time-consuming preparation, a particular fluorescence microscope, and skill in interpreting the results [43,49,50,51].

#### 5.1.3. Culture Techniques

The microbiological culture method can diagnose *H. pylori* infections with less sensitivity but more specificity (it reaches 100% specificity) than other methods. This approach has also been found to provide evidence of active infection, which is advised whenever treatment fails, and is a method for identifying fluoroquinolone- and clarithromycin-resistant *H. pylori* [97,98]. Depending on the therapeutic approach and the resistance to the therapy, this technique can be performed in laboratories that are well equipped, either as part of a scientific study or after the therapeutic approach fails to identify a resistance. *H. pylori* infection is diagnosed according to its phenotypic characteristics, Gram staining, biochemistry (e.g., urease, catalase, and oxidase activities), and peptide mass fingerprinting technology if available in the laboratory. The performance of antimicrobial sensitivity tests depends on several variables, including diagnostic specimen standards, the duration of transportation, aerobic conditions, and the quality of biopsy samples [99,100,101]. Furthermore, low bacterial counts, proton pump inhibitor use, antibacterial use, alcohol consumption, and haemorrhage are factors that impact the frequency of culture positivity. It is necessary to take at least two biopsy samples from the lower section and two from the middle section of the stomach, and antimicrobial medications must be discontinued for a minimum of four weeks before biopsy and subsequent culture can be performed [102].

### 5.2. Noninvasive Tests

#### 5.2.1. Stool Antigen Tests (SATs)

Using enzyme immunoassays or immunochromatography, one can determine whether saliva, blood, or stool contains an antigen induced by *H. pylori* infection. There is no doubt that an SAT is an extremely useful diagnostic tool for detecting and confirming bacterial persistence after treatment, with an overall accuracy of over 90% [21]. There is a tendency among physicians and patients to favour an SAT regardless of the patient’s condition since it is less expensive than other treatments [103]. As advised by SAT guidelines, the doctor should evaluate the eradication of the infection at least 4 weeks after the course of eradication medication has ended. The procedure is straightforward, but it is recommended that patients refrain from using proton pump inhibitors, antimicrobials, and bismuth agents for two weeks prior to screening. It is possible to conduct diagnostic and efficacy evaluations of eradication therapy by utilizing an SAT with monoclonal antibodies [93]. It is possible to use these tests for both adults and children following stomach surgery. It might be possible to prevent gastric neoplasia in the future through the use of these tests [103].

An SAT is often used as it is a better alternative to invasive procedures in the diagnosis of active *H. pylori* infections [104,105]. In comparison to other noninvasive tests, for example, the UBT test, an SAT has the main advantages of being simple, having a fast turnaround time, and being inexpensive. It was reported in a systematic review and meta-analysis based on Gisbert et al.’s paper [106] that a monoclonal SAT can be helpful for the diagnosis of *H. pylori* infection. Over the course of 22 clinical studies involving 2499 patients, a monoclonal SAT was used before eradication therapy was administered. As a result, the sensitivity and specificity were pooled in 94% and 97% of the cases, respectively. According to the manufacturer’s cut-off for the diagnosis of *H. pylori* infection in children, the monoclonal SAT used was found to have 100% sensitivity and 76.2% specificity for the diagnosis [107].

Although SATs are known to have many advantages, a number of drawbacks have also been identified and are worth considering. SAT results may vary across geographical regions due to the different antigens used for SATs in each area, since SATs rely on an antigen-antibody reaction to produce the results [108,109]. There is the possibility that a negative SAT result may not always reflect the absence of *H. pylori* infection owing to a limited bacterial colonization in the stomach and a low concentration of *H. pylori* antigen in the specimen [110]. Moreover, SATs may also be less sensitive in some extraordinary situations, including those involving patients experiencing abdominal bleeding or receiving bismuth-based medications [111]. Despite widespread acceptance, SATs remain burdensome and unhygienic, and patients dislike the idea of handling samples of faeces. The submission of a stool sample is also of primary importance when using this test in epidemiological research, particularly in areas without freezing equipment and especially in areas with limited access to standard laboratory equipment [112]. The stool samples should be kept at low temperatures (between −5 and −25 °C) (above seven days) if they are not examined within a short period of time. A temperature of −80 °C is recommended for any samples that will be stored over a long period of time to keep the antigen stable [113].

#### 5.2.2. Urea Breath Test (UBT)

Among noninvasive diagnostic tools, the UBT measures the ratio of carbon-13/14 isotopes (^13^C/^14^C) in exhaled air before and after the consumption of radioactive urea based on *H. pylori* urease activity. By converting urea to ammonia, urease, which is secreted by *H. pylori*, balances the pH of the stomach, allowing it to pass through mucus and attach to its cells. During the month prior to the test, proton pump inhibitors and antimicrobial treatment should be discontinued [114]. In brief, prior to administering^13^C-labelled urea to the patient, two samples must be collected. For the test to be as accurate as possible, the patient should fast for at least six hours, ideally overnight. Tubes or bags are first used to collect two breath samples. Following weighing, the patient is administered the diluted ^13^C-labelled urea solution. The next 30 min are used to collect two additional breath samples. A 100-mL sample of orange juice should be consumed by children between 5 and 12 years of age.

The active material in UBTs contains ^13^C-labelled urea instead of the more common ^12^C. *H. pylori* produces urea by converting it into carbon dioxide through its ureases. During the test, patients are exposed to ^13^C-labelled urea and exhale carbon dioxide containing ^13^C. The tagged carbon dioxide can be analysed with laser-assisted ratio analysers, infrared spectroscopy that is not dispersive, or isotope ratio mass spectrometry. Positive results are determined if carbon dioxide can be detected in the respiratory sample 30 min after collection. This technique can be beneficial for adults as well as children aged 3–11 [114,115]. One of the beneficial noninvasive investigative techniques is UBT in a “test-and-treat” approach. Contrary to serological and stool antigen tests, UBTs can be successfully used in patients after gastrectomy, antibacterial use, or recent proton pump inhibitor use.

#### 5.2.3. Serological Testing

At present, enzyme-linked immunosorbent assays (ELISAs) are used to measure antibody levels against *H. pylori* immunoglobulin G (IgG). Widely viable serological assays are frequently utilized in diagnostic settings. The specificity and sensitivity of these tests are 85% to 95%, respectively. IgG antibodies are produced during *H. pylori* infection and remain elevated for approximately a year before they return to normal levels. Serological testing fails to distinguish between recent and previous infections because antibodies are able to persist for years post-infection, which is why it is ineffective in determining posttherapy eradication rates [116,117]. Although serological screenings are sensitive between 55.6% and 100% and specific between 59.6% and 97.9%, they are not helpful in areas where infection rates are low.

### 5.3. Molecular Tests

#### 5.3.1. PCR and Real-Time PCR Testing

PCR or real-time PCR testing can detect *H. pylori* in stomach biopsy, digestive fluid, saliva, dental plaque, and stool samples. As a result of its high specificity and sensitivity, PCR testing is an excellent method to detect *H. pylori* in a quick and safe manner. PCR testing to confirm *H. pylori* cure is comparable, if not better than, culture techniques [118]. The PCR-based restriction fragment length polymorphism (PCR-RFLP) method can be applied to distinguish among *H. pylori* subtypes. For instance, 141 biopsy samples from 131 patients were utilized to identify and characterize the *H. pylori Urease-C* gene. Other conventional diagnostic techniques are not able to identify spiral or coccoid strains of *H. pylori*, which is one of the most important benefits of in-house PCR assays. PCR also has the advantage of being able to analyse DNA extracted from urease test samples sent via mail without strict transportation requirements. Numerous investigations have reported poor sensitivity identification in faecal specimens due to the low copy number of target DNA and PCR inhibitors [119]. Despite the clinical utility of PCR testing, several constraints restrict its use, such as time constraints, low output, and infection risks. In developing countries, PCR techniques are highly dependent in terms of a variety of factors, such as cost, equipment availability, and expertise. In the coming years, one of the most promising molecular methods will be real-time PCR sample hybridization, which can rapidly assess clarithromycin tolerance in biopsy and faeces specimens with high sensitivity and specificity using fluorescence resonance energy transfer probes [25,120,121].

#### 5.3.2. Peptide Mass Fingerprinting Technology

Peptide mass fingerprinting (PMF) technology can be used to detect and eradicate a variety of microorganisms with an effective, inexpensive, and accurate technique known as “MALDI-TOF MS” [122,123,124,125,126]. This method is advantageous because of its speed and cost savings. It is also highly accurate and sensitive. It can differentiate between different *Helicobacter* species [122] and can detect antibiotic resistance. Microbial biomass in small amounts (approximately 105 CFU) is also needed for the analysis. In recent years, PMF has become more rapidly accessible, more affordable, and more accurate than what was possible previously [125,127]. The identification of microorganisms using this technology is easy, and it has become an integral part of clinical laboratories in microbiology [128,129]. According to the provided protein sequences of each bacterial population, an ultraviolet laser is used to disintegrate and lyse the microbial biomass encased in a matrix (cyano-4-hydroxycinnamic acid). MALDI-TOF MS analyses particles by ionizing them, sorting them by mass-to-charge ratios, and recording their arrival times at detectors [130]. A complete *Helicobacter* library has been built utilizing MALDI-TOF MS by discovering 93 gastric Helicobacter isolates from ten different *Helicobacter* species [122]. To examine the variety of spectra within the same species and to differentiate between them, special software (Compass Explorer 4.1) was used to create a main spectrum library (MSP) dendrogram. Bruker’s recommendations do not allow accurate identification of genus (log score 1.70) or species (log score 2) using the most recent Microflex LT library. Once the internal *Helicobacter* library was finished, the second-best match was used for species identification [21].

As multiple drug-resistant strains of bacteria are becoming more prevalent, it is becoming increasingly important to establish reliable and effective methods of testing antibiotic susceptibility [131]. It has therefore been demonstrated in multiple studies that MALDI-TOF MS can be used to quickly detect antimicrobial resistance in various diseases as well as to identify dangerous fungi that are resistant to antimicrobials [131]. Accordingly, MALDI-TOF MS has been demonstrated to be effective in the management of bacterial infections, to support demographic research, and to aid in the creation of prevention and control programmes [132]. This method has several drawbacks despite its ability to accurately identify almost all species of microorganisms commonly seen in clinical laboratories. Considering how similar microorganisms are to each other, this method might not be able to distinguish between similar species. The biggest drawback is the need to culture bacteria before examination, which slows down the evaluation process significantly. The isolation and in vitro growth of Helicobacter species are well-known challenges [133]. The integrity of the MALDI-TOF MS spectrum has also been shown to be affected by culture duration [125]. Alternatively, bacterial ecology and protein expression patterns can be influenced by increasing or decreasing the growing medium [134].

## 6. The Prevention and Control of Multidrug-Resistant *H. pylori*

**Figure 3:**
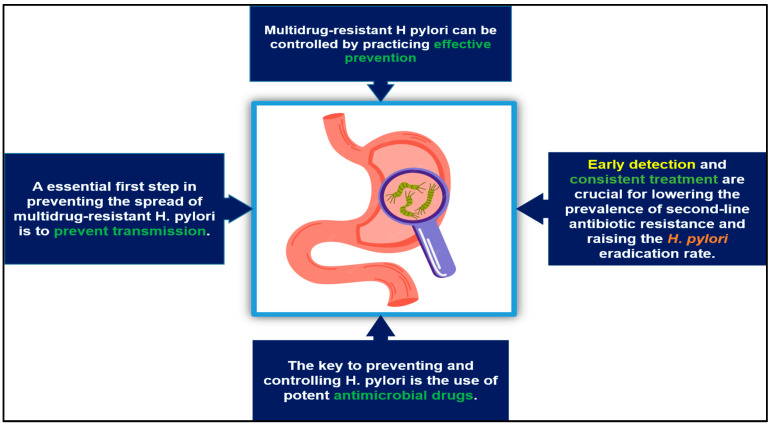
Prevention and control strategies of multidrug-resistant *H. pylori* infection.

Public health and the environment are at risk because of the high prevalence of infection with *H. pylori* and the extent of its pharmaceutical treatment [135]. Efforts should be made to find alternative methods of treating and preventing *H. pylori* infection. Strategies for preventing and treating multidrug-resistant *H. pylori* infection are provided in Figure 3, which can serve as a guide for the eradication of multidrug-resistant *H. pylori*. To combat multidrug-resistant *H. pylori*, current tactics include making an accurate diagnosis and providing consistent treatments, using antimicrobial drugs judiciously, and preventing *H. pylori* spread [136,137]. The control of multidrug-resistant *H. pylori* infections can be achieved through preventive measures. By implementing appropriate prophylaxis, infection can be reduced effectively. Identifying and treating drug-resistant infections efficiently and consistently are crucial for limiting recurrence and increasing *H. pylori* clearance rates. Whether invasive or noninvasive, diagnostics are the key to quick and accurate identification [138].

To create logical, precise therapeutic interventions, antimicrobials should be selected according to the resistance of microorganisms to antimicrobials [139]. Potent antibiotics play a critical role in avoiding and treating *H. pylori*. Initially, proton pump inhibitors are used in conjunction with two or three drugs for three to fourteen days to treat diagnosed *H. pylori* infections [140]. In various important protocols [139,140,141,142,143], regional antimicrobial resistance rates are crucial factors in determining which therapy to use first. It is also advised to avoid reusing pharmaceutical drugs from first therapies in later therapies since this could lead to a build-up of antibiotic resistance [144]. It is noteworthy that, even if resistance emerges, it can be managed by adjusting dosages and adding bismuth, allowing the reuse of an antibiotic after it has failed once [144].

Despite unclear guidelines on sensitivity testing for first-line treatment of *H. pylori*, testing is recommended in refractory cases. As a result of the correct diagnosis and complete eradication of *H. pylori*, the rates of stomach cancer and long-term peptic ulcer disease have significantly decreased [145,146]. There are several types of first-line treatments, including clarithromycin-based triple therapy (CTT), bismuth-based quadruple therapy (BQT), and nonbismuth-based quadruple therapy (NBQT) [147]. Antimicrobial resistance and low levels of eradication are associated with CTT. CTT should be selected based on the local prevalence of clarithromycin resistance as well as the history of macrolide usage [148]. If the local eradication rate is between 80 and 85%, the CTT resistance rate is higher than 15%, or clarithromycin tolerance is unknown, then preventive measures should be taken [141,149]. It is worth noting, however, that culturing or PCR testing can also be used to assess clarithromycin sensitivity, although this is less common in individuals or regions with low clarithromycin tolerance rates (15%) [146]. When the clarithromycin resistance rate is more than 15% or the clarithromycin resistance rate is in a region unknown, BQT should be used as a first-line treatment. As an initial treatment, BQT is recommended in regions where metronidazole and clarithromycin resistance rates are high. If BQT is not available, NBQT might be considered instead [146].

Antimicrobial resistance is widespread among World Health Organization (WHO) countries. It is reported that more than 15% of people in all WHO countries are resistant to levofloxacin, metronidazole, and clarithromycin. In many cases of eradication failure, antibiotic resistance is a major factor [146]. Several studies have linked clarithromycin resistance to the failures of treatments containing clarithromycin. A study based on the European Database on *H. pylori* Care found that 21,533 patients receiving CTT were resistant to clarithromycin before treatment, 32% were resistant to metronidazole, and 13% were resistant to both before treatment. A 90.5% eradication rate can be achieved only with BQT, the only method that has achieved an adjusted eradication rate of 81.5% in an intention-to-treat analysis. Other factors can affect eradication, including drug absorption, efflux, biofilm formation, and mutations [150]. Several drugs have been associated with drug efflux or uptake, including amoxicillin, levofloxacin, nitroimidazoles, and tetracyclines [150]. Tetracycline, clarithromycin, and amoxicillin are among the medications to which *H. pylori* is resistant owing to the formation of biofilms [151]. Coccoid bacteria with high cholesterol and fatty acid concentrations have been found to be resistant to antibiotics [152]. These resistance mechanisms can also be linked to the inappropriate use of antibiotics [152]. There is a higher eradication failure rate with clarithromycin-containing regimens among patients with a longer history of macrolide use and those with higher macrolide prescription rates.

Government, health care administration agencies, drug control agencies, and hospital professionals must all work together to improve the management of antimicrobial drug prescription [139,153]. Moreover, the establishment of a system for controlling sensible drug usage, the formulation of medication recommendations, the establishment of networks for tracking antibiotic resistance and antimicrobial drug standards, and encouraging the development of new drug classes are all crucial elements to the success of programmes to manage antimicrobial drug resistance. Furthermore, blocking transfer is crucial for stopping the spread of *H. pylori* if it cannot be treated [154]. Hospitals and laboratories need to tighten safety precautions to prevent drug-resistant viruses from spreading. In addition to blocking transfer, stopping the spread of *H. pylori* that cannot be treated is a crucial step. Safety precautions in hospitals and laboratories need to be strengthened to prevent the spread of drug-resistant bacteria [135,155].

There are several ways in which the public’s awareness of and attitudes towards *H. pylori* screening can be used to improve the development of effective *H. pylori* prevention and screening techniques. Teng et al. [156] and Wang et al. [157] conducted cross-sectional studies in which it was found that only a small number of participants had ever undergone an *H. pylori* test, and the majority knew little to nothing about this infection. Nevertheless, most participants expressed support for screening for *H. pylori*. The fact that testing is not performed regularly and that the benefits of testing are not sufficiently understood are the major factors deterring people from getting tested for *H. pylori* infection. In addition, six studies provided information about *H. pylori* [158,159,160,161,162,163]. There was a lack of knowledge about *H. pylori* in the general public based on all investigations. As part of two studies conducted in which people were asked if they had heard of *H. pylori*, only 22–35% of those asked said yes to the question [158,159]. Unexpectedly, one study found that people who tested negative for *H. pylori* were significantly more likely to have heard of it than those who tested positive [159]. According to Driscoll and his co-workers [164], seven studies have been performed on *H. pylori* prevention methods [158,159,160,165,166,167,168] in the last few years. A healthy lifestyle, good hand washing habits, and safe food handling techniques all contributed to fewer *H. pylori* infections [164].

## 7. Recent Advancements in Diagnostics and Treatment

It has been over a decade since *H. pylori* was first detected, and in the last few years, immense advances have been made in both diagnostic and therapeutic methods for the treatment of patients infected with *H. pylori*. The development of nanoparticles over the last few years has been one of the most exciting developments in the field of therapy and diagnosis, and nanoparticles have the potential to assist in the replacement of expensive and invasive endoscopic procedures with no-cost, less invasive alternatives in the near future [169,170]. In this regard, the use of a biosensor is one of these technologies, as a biosensor will be able to produce audible signals by converting distinctive biological components connected to a transducer surface [171,172]. Despite the fact that this method is performed in a more straightforward manner than other techniques, such as PCR testing or immunoassays, it still provides precise and accurate results and allows the accurate diagnosis of disorders [172,173]. Either the antibody against *H. pylori* or the antigen against the bacterium must be identified during this complicated procedure. Piezoelectric materials detect illness through alterations in acoustics, sensor arrays detect illness with changes in fluorescence or colour absorption, and thermal sensors detect illness with changes in temperature [173]. The adherence of a transducing element to a transducing surface alters its electromotive force or conductance. The unique use of single-stranded genomic DNA patterns, which are highly tuned to specific antigens, peptides, or antibodies, by Yadav et al. [174] has been discussed with high expectations for their potential therapeutic application [174].

There are recent therapeutic developments that utilize nanotechnology to enhance medication transport and to have an immediate antimicrobial effect [175]. *H. pylori* therapy may be enhanced through the creation of novel medications. The prokinetic drug vonoprazan (VPZ) is an acid blocker that is potassium-competitive, which makes it an effective alternative to proton pump inhibitors for classic acid suppression. VPZ does not require stomach acid to work, and it has a prolonged half-life. In addition, variants of the CYP450 gene do not affect the activity of VPZ [176]. The relationship between slower CYP metabolizers and higher elimination levels has been suggested in several studies [176,177,178], although this has not always been clinically meaningful. It has been shown that variants in genes involved in the immune system response can have similar effects on illness severity and complication risk [179,180,181]. In fact, VPZ has only recently become available to patients in Asian countries, and multiple meta-analyses have demonstrated that it is more effective than conventional triple therapy containing proton pump inhibitors [182,183,184]. VPZ has been shown to exhibit equivalent and even better elimination outcomes than proton pump inhibitors in RCTs implementing a variety of implementation strategies and across low-resistance and high-resistance areas for clarithromycin, according to the most recent meta-analysis of RCTs [184]. Additionally, consumers of VPZ were reported to have more positive experiences with the product [184]. Antimicrobial peptides have a specific structure that damages the negative charge on the cell membrane, damaging the cell and interfering with the functionality of the cell [185]. Photodynamic treatment relies on photosensitive molecules produced by microbes that, in turn, create cytotoxic reactive oxygen species that kill bacteria. The use of bacteriophages specific to *H. pylori* induces cell lysis and eradicates the pathogen [186].

## 8. The Impacts of Compliance and Resistance on the Success of *H. pylori* Treatment

The two biggest obstacles in developing the most effective *H. pylori* treatment regimens are viewed by an overwhelming majority of medical professionals and researchers as increasing patient compliance and addressing antibiotic resistance [187]. It is imperative that patients receive sufficient education and information before they begin their *H. pylori* eradication treatment to ensure compliance, mainly because treatment for *H. pylori* eradication can be a complicated process and is associated with potential side effects [188]. A meta-analysis of the effectiveness of enhanced patient education programmes demonstrated significant increases in rates of medication adherence and eradication among participants [189]. The results of a project conducted in China that used social media as a means of educating, advising, and encouraging patients over the course of their therapy led to an eradication rate of 90% compared to 77% for the controls [190]. A comprehensive review was conducted this year using enhanced patient instructions, examples of which included a short-message service, telephone-based re-education, and the use of WeChat [191].

A survey of patients who received enhanced patient instructions showed not only a noticeable increase in the *H. pylori* eradication rate but also improved patient compliance and increased patient satisfaction compared to patients who received only conventional instructions. Similar results were observed in a meta-analysis of the patient compliance, eradication rate, and technology-enhanced communication tactics of similar programmes [188], although the compliance and eradication rates were much higher. Due to the unprecedented challenge that microbiologists worldwide faced during the *H. pylori* outbreak, it comes as no surprise that there has been relatively little published research relating to the prevalence of *H. pylori* resistance. The results of an important meta-analysis of antibiotic resistance rates in Australia and New Zealand over the past twenty years, conducted in a geographical area not previously examined, indicated that clarithromycin resistance had more than doubled during that period, from 7.4% to 16.1%, with stable rates of resistance to metronidazole, fluoroquinolones, amoxicillin, and tetracyclines [192]. An interesting meta-analysis of *H. pylori* resistance patterns discusses the phenomenon of heteroresistance, which occurs when resistant and sensitive *H. pylori* populations coexist within the same sample and/or when sensitivity patterns vary between different biopsy samples in an intriguing manner [193]. According to 22 studies that evaluated 3852 patients who had positive results for *H. pylori*, clarithromycin heteroresistance developed in 6.8% of patients, and metronidazole heteroresistance developed in 13.8% [187].

## 9. Family-Based *H. pylori* Eradication Strategies

In comparison to population- or community-based approaches, the advantages of a whole-family approach are that it identifies and continues to treat people who are infected by *H. pylori* [194]. When there is a high rate of infection in a population, as well as in a family, whose members have a significant stake, it is likely that these individuals will be motivated to become involved. Taking advantage of this method improves the management and monitoring of infected patients so that precancerous lesions can be detected earlier [195]. The preliminary practice findings of a whole family-based approach suggest that patients and family members are highly satisfied with the treatment they receive and are highly compliant; therefore, this approach deserves further research and improvement [195]. A concern with this approach is that it can lead to an overscreening of involved family members, which can lead to overdiagnosis. Noninvasive serological tests, urease breath tests, and stool antigen tests are all more cost effective, accessible, and effective than invasive serological tests, so these approaches are viable alternatives for testing and treating entire families whose members are at high risk of developing *H. pylori* infection [194]. As a general rule, it is not recommended that individuals with asymptomatic infections of *H. pylori* be tested for infections, but in some cases, such as patients who require long-term nonsteroidal anti-inflammatory drug therapy or individuals with a family history of stomach cancer, this may be necessary [196,197]. Patients with dyspepsia-like symptoms should not be routinely tested for *H. pylori*, nor should empiric eradication treatment be prescribed without testing for the presence of *H. pylori* [198].

## 10. The Potential Use of Vaccines

Since the stomach mucosa constantly regenerates and the stomach pH is acidic, *H. pylori* can remain hidden from the immune response of the body [199]. There is no assurance of ongoing safety even if *H. pylori* is completely eradicated [200]. A vaccine against *H. pylori* would reduce the frequency and severity of gastrointestinal illnesses as well as prevent or eradicate them [201]. It is important to choose a viable method to administer a preventive or therapeutic vaccine in combination with an efficient adjuvant and immunogenic bacterial antigens [202]. Several vaccine-related antigens can be found in vaccines, including *CagA*, *VacA*, *BabA*, *HpaA*, *NapA*, *OipA*, *GGT*, *HspA*, *Omp*, and *FliD* [203]. The CTB-UE [204] and FVPE [205] vaccines contain antigens and adjuvants that contain epitopes expressed on CD4+ and CD8+ cells. To boost immunogenicity, cholera toxin and *Escherichia coli* enterotoxin have been used as mucosal adjuvants in the development of several vaccines, including whole-cell or subunit vaccines. Additionally, the use of intramuscular *H. pylori* subunit vaccines with aluminium hydroxide adjuvants and oral delivery of live vector vaccines expressing *H. pylori* antigens is recommended to promote long-lasting protection (for example, *Salmonella* strains attenuated by attenuation and *Listeria monocytogenes* virulent strains) [206,207,208].

*H. pylori* vaccines failed to prevent microbial burden and provided only modest immunity in smaller animals and patients [209]. Vaccines are primarily in the preclinical or phase I stages, lack consistency, and produce variable results [203]. The results of a phase 3 randomized study, however, showed that oral vaccines with recombinant urease B were safe and effective in children [210]. Future policy looks promising and predictable. Plausible alternative treatment options in addition to vaccines are being developed. Studies have demonstrated that both probiotics and prebiotics are effective as adjuvants for the treatment of *H. pylori* [211,212]. Several antimicrobial peptides have antibacterial activities against *H. pylori* [213], even those with multidrug-resistant forms of the organism. The properties of these peptides include their helical shape, cationic nature, high positive charge, and isoelectric point [214]. *H. pylori* drug resistance could be prevented if these compounds were used instead of antibiotics [214]. During photodynamic treatment, a photosensitizer produces reactive oxygen species that oxidize biomolecules and cause irreparable damage. It is possible to eradicate *H. pylori* with photodynamic treatment regardless of tolerance to drugs.

A photosensitizer that targets *H. pylori* can prevent unexpected phototoxicity to human cells. An *H. pylori*-focused photodynamic treatment technique using an endoscopic laser system has been proposed [215]. *H. pylori* is still being fought with bacteriophages, such as the use of particular lytic phages, which show promise in this fight. There have been studies showing that natural items, such as fruits and vegetables, spices, and herbal remedies, have antagonistic effects on *H. pylori*, suggesting that they might be a useful alternative to antibiotic therapy [216]. Vaccination is therefore a promising technique for preventing *H. pylori* infection worldwide. Numerous efforts have been made in the past to develop an *H. pylori* vaccine, but the results have been disappointing [217]. Despite some vaccine candidates showing promise for prophylactic use, none has been shown to be clinically applicable [217,218].

## 11. Conclusions

Throughout history, *H. pylori* has garnered a great deal of attention because it is a widespread and complicated pathogen. Gastric ulcers and cancer are serious side effects that can arise from *H. pylori* infection. Therefore, early detection, adequate follow-up, and alternative treatments are necessary. A thorough understanding of *H. pylori* infection pathogenesis has enabled us to identify diagnostic and therapeutic targets. In particular, we can improve surveillance and our understanding of bacterial reinfection and dissemination and better control outbreaks. However, we can still improve our knowledge, as there is more to learn. Regular sensitivity testing as well as retesting on a regular basis, meticulous endoscopic monitoring on a regular basis, and health promotion all contribute to reducing or eliminating these issues and improving *H. pylori* treatment. Combining alternative therapies with current antibiotic regimens is also a possibility. Increasing evidence suggests that *H. pylori* infection may play a role in an increasing number of extraintestinal disorders. Although most of them do not manifest during infancy, knowing that they exist is crucial to preventing *H. pylori* infection in children since inflammation associated with *H. pylori* can begin during early life. As a result, preventing the development of all of the aforementioned extraintestinal symptoms is possible only when the infection is detected early and eradicated effectively. The production of a vaccine could achieve a range of preventative and eradication effects.

## Data Availability

Not applicable.
